# Prevalence of comorbid mental and physical illnesses and risks for self-harm and premature death among primary care patients diagnosed with fatigue syndromes

**DOI:** 10.1017/S0033291719001065

**Published:** 2020-05

**Authors:** Matthew J. Carr, Darren M. Ashcroft, Peter D. White, Nav Kapur, Roger T. Webb

**Affiliations:** 1 Division of Psychology and Mental Health, School of Health Sciences, Faculty of Biology, Medicine and Health, University of Manchester, Manchester Academic Health Science Centre, Manchester M13 9PL, UK; 2 NIHR Greater Manchester Patient Safety Translational Research Centre, Manchester M13 9PL, UK; 3 Division of Pharmacy and Optometry, Centre for Pharmacoepidemiology and Drug Safety, School of Health Sciences, Faculty of Biology, Medicine and Health, University of Manchester, Manchester Academic Health Science Centre, Manchester M13 9PT, UK; 4 Wolfson Institute, Barts and the London School of Medicine and Dentistry, Queen Mary University of London, London, UK; 5 Greater Manchester Mental Health and Social Care Trust, Manchester, UK

**Keywords:** Co-morbidities, fatigue syndrome, premature mortality, self-harm, suicide

## Abstract

**Background:**

Fatigue syndromes (FSs) affect large numbers of individuals, yet evidence from epidemiological studies on adverse outcomes, such as premature death, is limited.

**Methods:**

Cohort study involving 385 general practices in England that contributed to the Clinical Practice Research Datalink (CPRD) with linked inpatient Hospital Episode Statistics (HES) and Office for National Statistics (ONS) cause of death information. A total of 10 477 patients aged 15 years and above, diagnosed with a FS during 2000–2014, were individually matched with up to 20 comparator patients without a history of having a FS. Prevalence ratios (PRs) were estimated to compare the FS and comparison cohorts on clinical characteristics. Adjusted hazard ratios (HRs) for subsequent adverse outcomes were estimated from stratified Cox regression models.

**Results:**

Among patients diagnosed with FSs, we found elevated baseline prevalence of: any psychiatric illness (PR 1.77; 95% CI 1.72–1.82), anxiety disorders (PR 1.92; 1.85–1.99), depression (PR 1.89; 1.83–1.96), psychotropic prescriptions (PR 1.68; 1.64–1.72) and comorbid physical illness (PR 1.28; 1.23–1.32). We found no significant differences in risks for: all-cause mortality (HR 0.99; 0.91–1.09), natural death (HR 0.99; 0.90–1.09), unnatural death (HR 1.00; 0.59–1.72) or suicide (HR 1.68; 0.78–3.63). We did, however, observe a significantly elevated non-fatal self-harm risk: HR 1.83; 1.56–2.15.

**Conclusions:**

The absence of elevated premature mortality risk is reassuring. The raised prevalence of mental illness and increased non-fatal self-harm risk indicate a need for enhanced assessment and management of psychopathology associated with fatigue syndromes.

## Introduction

The term ‘fatigue syndrome’ (FS) describes a set of debilitating illnesses that affect large numbers of individuals and can greatly restrict the quality of life. For example, chronic fatigue syndrome (CFS) has been reported as affecting between 0.4% and 1.7% of the population internationally (Skapinakis *et al*., 2003; Lorusso *et al*., 2009; Johnston *et al*., 2013). According to current UK guidance on the diagnosis and management of CFS, a diagnosis should be considered if the onset of fatigue had a clear starting point, has lasted for several months and is not related to another condition such as anaemia, an underactive thyroid gland, liver or kidney disease (Baker and Shaw, 2007). Other symptoms may also be present including problems with sleeping and concentration, and muscle pain. Similar disorders include neurasthenia, post-viral fatigue syndrome (PVFS) and myalgic encephalomyelitis (ME). ME is often regarded as a synonym for CFS while PVFS is often considered to be either a synonym or a precipitant (Capelli *et al*., 2010; Moss-Morris *et al*., 2013; Brurberg *et al*., 2014).

The focus of many previous FS studies has either been on the potential causes or on attempts to define diagnostic criteria. While a large number of studies have reported on the prevalence of comorbid psychiatric disorders (Afari and Buchwald, 2003; Ranjith, 2005; Cella *et al*., 2013; Mariman *et al*., 2013; Daniels *et al*., 2017; Larkin and Martin, 2017; Williams *et al*., 2017), the prevalence of comorbid physical illnesses appears to have been largely neglected. Evidence regarding premature mortality risk is also comparatively sparse and inconclusive with some studies reporting risk elevations (Jason *et al*., 2006; Jason *et al*., 2011; McManimen *et al*., 2016), another reporting lower risk (Smith *et al*., 2006) and the largest study (*N* > 2000) showing no change in risk (Roberts *et al*., 2016), although the generalisability of the findings reported from the latter study was questionable because it was based on a single secondary care specialist service. Heightened risk has been noted in the few studies that have examined suicide, but these have been limited by a lack of statistical power (Jason *et al*., 2006; Kapur and Webb, 2016; McManimen *et al*., 2016; Roberts *et al*., 2016), and we were unable to find any published studies that have reported on the association between FSs and non-fatal self-harm risk. In general, evidence relating to adverse outcomes from large epidemiological studies is limited.

To address these gaps in the existing evidence-base we conducted a large cohort study among general practice-registered patients to estimate the prevalence of comorbid mental and physical illnesses, and risks of non-fatal self-harm, suicide and all-cause mortality, among patients diagnosed with a FS *v.* a large age-, gender- and practice-matched comparison cohort without a FS diagnosis. To minimise the impact of terminological disagreement, diagnostic uncertainty and likely variation in general practitioner (GP) coding practices, we opted to coalesce all FSs for our primary analyses, but also repeated our analyses in those diagnosed with CFS or ME as a sensitivity analysis. We hypothesised that the prevalence of both mental and physical comorbidities would be greater in the FS cohort (Afari and Buchwald, 2003; Ranjith, 2005; Cella *et al*., 2013; Daniels *et al*., 2017; Larkin and Martin, 2017; Williams *et al*., 2017). We estimated relative risks for all-cause mortality, natural and unnatural causes of death and non-fatal self-harm. In response to conflicting findings reported from previously published studies (Jason *et al*., 2011; McManimen *et al*., 2016; Roberts *et al*., 2016), we tested the specific hypothesis that suicide and self-harm risks would be elevated in patients with an FS diagnosis, but that all-cause mortality risk would not be greater than in the rest of the population without an FS.

## Methods

### Data sources

The study was conducted using electronic health data extracted from the Clinical Practice Research Datalink (CPRD) obtained under licence from the UK Medicines and Healthcare products Regulatory Agency (MHRA) (Herrett *et al*., 2015). The CPRD is one of the world's largest population-based, longitudinal, primary care databases, containing anonymised patient information provided by participating general practices. Diagnoses are coded using the Read system that is in standard usage in UK general practice. We also utilised linkages between the CPRD and external data sources. Inpatient Hospital Episode Statistics (HES) were used to augment capture of key risk factors and associated conditions, including comorbid mental and physical illness diagnoses, and also enhance ascertainment of non-fatal self-harm as an outcome. Data from the Office for National Statistics (ONS) were used to ascertain specific causes of death according to the 10^th^ revision of the International Classification of Diseases (ICD-10). Records were available for patients registered with 385 practices in England, constituting approximately 60% of all CPRD practices across the UK. These were the practices that were participating in the CPRD linkage scheme, linking data for all eligible patients with a valid National Health Service (NHS) identifier.

### Study cohort

Cohort members were aged 15 years and over, were registered in a CPRD practice, and were given the first diagnosis of any FS between 1 January 2000 and 31 March 2014. Using the Read codes listed in the Appendix, we defined the index date as the first occurrence of a relevant FS diagnostic code in a patient's medical record. We included patients who had been registered with an ‘up-to-standard’ (for research) practice for at least one year at that time. Follow-up ended when the patient either died, transferred out of the practice, the general practice stopped collecting data, the end of the study period (31 March 2014) or the outcome of interest occurred. Using incidence density sampling, a representative comparison cohort was created whereby each patient with an incident FS diagnosis was matched with up to 20 comparison patients without a FS to create a ‘matched set’. Requiring the comparison patients to have been unaffected by any form of FS at first diagnosis date, we matched the patients on age, gender and registered general practice. The same registration and practice CPRD contribution criteria were applied when sampling patients for the comparison cohort. Henceforth, we refer to these two patient cohorts as the ‘FS cohort’ and the ‘FS-free comparison cohort’.

We also identified a subset of patients nested within the broadly classified FS cohort consisting of patients with a definitive diagnosis of CFS or ME on their index date. In the code list provided in the Appendix, a column headed ‘CFS/ME’ distinguishes the Read codes used to identify patients diagnosed with CFS from those diagnosed with other types of FS or those with a diagnosis that was recorded using the ambiguous code ‘Fatigue syndrome’ (Eu46011). Henceforth, we refer to this sub-cohort as the ‘CFS sub-cohort’ and their respective matched comparison patients (without a fatigue syndrome diagnosis) as the ‘CFS-free comparison sub-cohort’.

### Baseline risk factors and diagnoses of comorbidities

Read code lists were developed to identify diagnoses of comorbid mental illness among cohort members prior to study entry. We grouped the diagnoses into six specific categories: depression, anxiety disorders, schizophrenia spectrum disorders, bipolar disorder, eating disorders and personality disorders. Prescriptions for psychotropic medications were extracted from the database using Multilex product codes for antidepressants, antipsychotics and anxiolytics/hypnotics. Read code lists were also constructed for: all physical conditions that feature in the Charlson comorbidity index (Charlson *et al*., 1987), alcohol misuse or dependence and smoking status. For both mental and physical illnesses, we augmented the identification of diagnoses recorded in primary care with ICD-10 coded HES data. All coding lists applied in the study are accessible from the clinical codes repository (http://www.clinicalcodes.org) (Springate *et al*., 2014).

### Adverse outcomes

The outcomes examined were all-cause mortality, natural deaths, unnatural deaths, suicide and non-fatal self-harm. Causes of death were defined according to established ICD-10 classification ranges. Following accepted practice for UK-based epidemiological research, we included ‘open verdicts’ in our suicide definition (Linsley *et al*., 2001). To identify self-harm episodes, we used the definition ‘any act of self-poisoning or self-injury, irrespective of the apparent purpose’ as used in UK National Institute for Health and Care Excellence (NICE) guidelines (National Institute for Health and Care Excellence, 2011). This approach is based on the notion that attempting to distinguish between self-harm episodes involving suicidal intent *v.* non-suicidal self-injury creates a false dichotomy as some individuals are not easily allocated to a single category due to the choice of method for a specific episode and/or method-switching over time (Kapur *et al*., 2013). Using this broad conceptualisation we developed a list of Read codes to delineate all self-harm episodes across the spectrum from milder forms of non-suicidal behaviour through to near-fatal suicide attempts.

### Statistical analyses

All analyses were performed using Stata software version 13 (StataCorp LLC). Deprivation was measured according to patient postcodes using the Index of Multiple Deprivation (IMD) 2010 quintiles. The IMD provides a means of ranking and assessing whether a locality is more or less deprived than others (Department for Communities and Local Government, 2011). Prevalence ratios (PRs) were used to compare the clinical characteristics and baseline risk factors for the FS cohort *v.* the matched FS-free comparison cohort on entry into the study. At baseline, we examined diagnoses of comorbid mental and physical illnesses, psychotropic medication prescription, histories of self-harm and alcohol misuse, and smoking status. The PRs were estimated using conditional Poisson regression with fixed effects on the matched sets and robust variance estimation (Barros and Hirakata, 2003; Tamhane *et al*., 2016). Prevalence is the proportion of people with a specified risk factor, among a specified population and at a specified time (the index date). The PRs in our study provide a measure of association between the exposure (i.e. a diagnosed FS) and, in our context, a baseline risk factor or co-morbidity. The PR is calculated by dividing the prevalence in the FS cohort by the prevalence in the FS-free comparison cohort. A PR that is statistically significantly greater than unity indicates a positive association; i.e. the prevalence among the FS cohort is greater than the prevalence among the FS-free comparison cohort. For the adverse outcomes, we compared the event rates for the two cohorts and conducted stratified Cox regression survival analyses. The estimated hazard ratios (HRs) were adjusted for the potential time-dependent confounding effects of alcohol misuse and smoking status. The proportional hazards assumption was then formally assessed using the Grambsch–Therneau test (Grambsch and Therneau, 1994) and graphical diagnostics were performed based on the scaled Schonfeld residuals (Schoenfeld, 1982). Finally, we replicated the analytical process to compare the CFS and CFS-free comparison sub-cohorts.

## Results

The demographic breakdown of patients in the FS cohort is summarised in Table 1. Nearly three-quarters of the cohort were female and the median age at diagnosis was 43 (IQR 24). We observed a gradient of decreasing numbers of individuals diagnosed in more deprived areas. Table 2 documents the numbers, percentages and PRs for an array of baseline risk factors on cohort entry. We observed a greater prevalence of mental illness diagnoses in the FS cohort *v.* the FS-free comparison cohort. This finding was consistent across all diagnostic categories examined apart from schizophrenia spectrum disorders, although the absolute difference in prevalence between the FS and comparison cohorts was only substantial (i.e. >2% difference) for two categories: anxiety disorders and depression. We found a substantial overlap in the number of diagnoses of anxiety disorders and depression. Among patients with a prior anxiety disorder diagnosis, 53% of patients in the FS cohort and 44% in the FS-free comparison cohort had also had a prior depression diagnosis. Consistent with the findings on psychiatric diagnoses, the prevalence of psychotropic medication prescribing was elevated in the FS cohort. Histories of self-harm and of alcohol misuse were also more prevalent in the FS cohort, although the difference for the latter risk factor was non-significant. In contrast, the proportion of current or ex-smokers was lower, but, although the estimated difference was significant, its magnitude was small and the FS-free comparison cohort also contained a greater proportion of patients with unknown smoking status.

**Table 1.** Demographic characteristics of the FS cohort and FS-free comparison cohort

		FS cohort		FS-free comparison cohort	
		*N*	%	*N*	%
All persons		10 477		209 402	
Gender					
	Female	7477	71.4	149 454	71.4
	Male	3000	28.6	59 948	28.6
Age in years					
	15–34	3276	31.3	65 510	31.3
	35–54	4554	43.5	91 059	43.5
	55 and over	2647	25.3	52 833	25.2
IMD quintile					
(Least deprived)	1	2566	24.5	52 424	25.0
	2	2480	23.7	49 717	23.7
	3	2265	21.6	43 045	20.6
	4	1826	17.4	36 471	17.4
(Most deprived)	5	1340	12.8	27 745	13.2

FS, fatigue syndrome; IMD, Index of Multiple Deprivation.

**Table 2.** Comparison of baseline prevalence of risk factors and co-morbidities in the FS cohort *v*. its matched FS-free comparison cohort

	FS cohort (*N* = 10 477)		FS-free comparison cohort (*N* = 209 402)			
	*n*	%	*n*	%	PR	95% CI
Co-morbid mental illnesses:						
Schizophrenia spectrum	73	0.7	1445	0.7	1.01	(0.80–1.28)
Bipolar disorder^a^	85	0.8	1047	0.5	1.62	(1.30–2.02)
Depression^a^	3478	33.2	36 771	17.6	1.89	(1.83–1.96)
Anxiety disorder^a^	3507	33.5	36 505	17.4	1.92	(1.85–1.99)
Eating disorder^a^	199	1.9	2120	1.0	1.88	(1.62–2.17)
Personality disorder^a^	127	1.2	953	0.5	2.66	(2.21–3.20)
Any of the above diagnostic groups^a^	5200	49.6	58 751	28.1	1.77	(1.72–1.82)
Psychotropic medication prescriptions						
Anti-depressant drugs^a^	5331	50.9	54 158	25.9	1.97	(1.91–2.02)
Anti-psychotic drugs^a^	2224	21.2	23 637	11.3	1.88	(1.80–1.96)
Anxiolitics and hypnotics^a^	3193	30.5	36 712	17.5	1.74	(1.68–1.80)
Any of the above medication types^a^	6433	61.4	76 466	36.5	1.68	(1.64–1.72)
History of self-harm^a^	563	5.4	7255	3.5	1.55	(1.42–1.69)
History of alcohol misuse/dependence	159	1.5	2791	1.3	1.14	(0.97–1.34)
Smoking history^b^						
Current/ever	4309	41.1	87 006	41.5	0.96	(0.93–0.99)
Never	6026	57.5	113 517	54.2		
Unknown	142	1.4	8879	4.2		
Co-morbid physical illness^a^	3523	33.6	55 138	26.3	1.28	(1.23–1.32)

PR, prevalence ratio; CI, confidence interval.

Co-morbid physical illness determined via the Charlson index.

a Denotes a PR that is significantly >1.

b Denotes a PR that is significantly <1.

The proportion of patients diagnosed with one or more comorbid physical illnesses, as determined using the list of Charlson index conditions, was greater in the FS cohort, although the PR and the magnitude of the difference between cohorts (7.3%; 95% CI 6.4–8.2%) was considerably smaller than that observed for one or more mental illness diagnoses (21.6%; 20.6–22.6%). Table 3 presents comparisons of prevalence for specific comorbid physical illnesses between the FS cohort and its comparison cohort on entry into the study. With the exception of dementia and metastatic tumours, both of which had very low prevalence values in both cohorts, the prevalence of all comorbid physical illnesses examined was the same or greater in the FS cohort. Chronic pulmonary disease was by far the most prevalent comorbid physical condition in both cohorts. Each Charlson index condition is allocated a score (weight) based on the 1-year risk of mortality, and the index is calculated as the sum of the scores over all the conditions. Table 4 summarises the patient weighted ‘scores’ calculated using the Charlson comorbidity index. We found a significant difference in the distribution of the scores for the FS cohort *v.* its comparison cohort, with group differences dominated by patients with either an absence of comorbidity or a single diagnosed illness.

**Table 3.** Comparison of the baseline prevalence of co-morbid physical illnesses in the FS cohort *v*. its matched FS-free comparison cohort

	FS cohort (*N* = 10 477)		FS-free comparison cohort (*N* = 209 402)			
Charlson condition	*n*	%	*n*	%	PR	95% CI
AIDS	<5	–	25	0.0	–	–
Cancer	368	3.5	6992	3.3	1.05	(0.95–1.17)
Cerebrovascular disease^a^	193	1.8	3044	1.5	1.26	(1.09–1.46)
Chronic pulmonary disease^a^	2363	22.6	34 721	16.6	1.36	(1.30–1.42)
Congestive heart disease	82	0.8	1669	0.8	0.98	(0.78–1.22)
Dementia^b^	9	0.1	555	0.3	0.32	(0.17–0.62)
Diabetes	337	3.2	6693	3.2	1.01	(0.90–1.12)
Diabetes with complications	69	0.7	1372	0.7	1.00	(0.79–1.28)
Hemiplegia	42	0.4	620	0.3	1.35	(0.99–1.85)
Metastatic tumour	32	0.3	740	0.4	0.86	(0.61–1.23)
Liver disease^a^	61	0.6	809	0.4	1.59	(1.22–2.07)
Myocardial infarction	119	1.1	2207	1.1	1.08	(0.89–1.29)
Peptic ulcer disease^a^	260	2.5	3588	1.7	1.45	(1.28–1.64)
Peripheral vascular disease^a^	119	1.1	1889	0.9	1.26	(1.04–1.51)
Renal disease^a^	209	2.0	3109	1.5	1.34	(1.17–1.54)
Rheumatological disease^a^	244	2.3	3281	1.6	1.49	(1.30–1.69)

PR, prevalence ratio; CI, confidence interval.

Values omitted for AIDS due to small numbers.

Mild and moderate liver disease coalesced in a single category due to small numbers.

a Denotes a PR that is significantly >1.

b Denotes a PR that is significantly <1.

**Table 4.** Comparison of distributions of Charlson Index co-morbidity scores in the FS cohort *v*. its matched FS-free comparison cohort

Charlson	FS cohort (*N* = 10 477)		FS-free comparison cohort (*N* = 209 402)			
Index	*n*	%	*n*	%	χ_5_^2^	*p* value
0	6954	66.4	154 264	73.7	287.9	<0.001
1	2589	24.7	39 044	18.7		
2	523	5.0	8885	4.2		
3	208	2.0	3866	1.9		
4	87	0.8	1380	0.7		
⩾5	116	1.1	1963	0.9		

FS, fatigue syndrome.

The adjusted HRs for subsequent adverse outcomes during follow-up are presented in Table 5. We did not find elevated risk for the FS cohort in relation to all-cause mortality or all natural *v.* all unnatural causes of death. Although the HR for suicide was greater than one, the absolute event rates and the difference between the rates were modest, and the risk elevation was not statistically significant. However, we did find a significant difference in non-fatal self-harm risk, for which the event rate for the FS cohort was nearly twice that observed for its comparison cohort: adjusted HR 1.83 (1.56–2.15).

**Table 5.** Hazard ratios comparing risks of premature mortality and non-fatal self-harm in the FS cohort *v*. its matched FS-free comparison cohort

	FS cohort		FS-free comparison cohort		HR	(95% CI)
	*n*	Rate/1000 PY	*n*	Rate/1000 PY		
All deaths	464	7.20	9240	7.25	0.99	(0.91–1.09)
Natural deaths	450	6.98	8969	7.04	0.99	(0.90–1.09)
Unnatural deaths	14	0.22	271	0.21	1.00	(0.59–1.72)
Suicide	7	0.11	81	0.06	1.68	(0.78–3.63)
Self-harm^a^	163	2.55	1748	1.38	1.83	(1.56–2.15)

HR, hazard ratio.

HRs adjusted for alcohol misuse and smoking status.

a Denotes an HR that is significantly >1.

In online Supplementary Tables S1–S3, we present the findings for the nested CFS sub-cohort and its comparison sub-cohort. The number of patients that received a definitive diagnosis of CFS (*n* = 4486) comprised less than half of the combined FS cohort and female patients accounted for 74.4% of the sub-cohort. Contrasting the CFS sub-cohort with its matched comparison sub-cohort, the PRs and their lower confidence intervals for prior mental illness diagnoses, including those in the schizophrenia spectrum, and histories of psychiatric medication prescriptions were all significantly greater than one and, in all cases, were greater than those found when comparing the combined FS cohort against its comparison cohort: see online Supplementary Table S1. Similarly, the baseline prevalence of prior self-harm and one or more comorbid physical illnesses was significantly elevated in the CFS sub-cohort, again to a greater extent than the elevated prevalence found for the combined FS cohort.

Online Supplementary Table S2 summarises the prevalence of comorbid physical illnesses in the CFS and CFS-free comparison sub-cohorts. We found notably elevated PRs for cerebrovascular disease, chronic pulmonary disease, diabetes with complications, hemiplegia, peptic ulcer disease and rheumatological disease. However, the elevated PRs were primarily attributable to smaller proportions of affected individuals in the CFS-free comparison sub-cohort than observed in the larger FS-free comparison cohort. The statistical analysis of adverse outcomes is presented in online Supplementary Table S3. In contrast with the findings for the combined FS cohort, we found a significantly reduced risk of all-cause mortality among the CFS sub-cohort *v.* its comparison sub-cohort: HR 0.76; 0.60–0.97. On the other hand, we found an increased non-fatal self-harm risk among the CFS sub-cohort: HR 2.11; 1.67–2.65. When comparing the CFS and CFS-free comparison sub-cohorts, the self-harm risk elevation was greater than that between the combined FS and FS-free comparison cohorts.

## Discussion

### Main findings

We found a raised prevalence of comorbid mental illnesses and psychotropic medication prescribing among patients diagnosed with a FS. Prevalence of anxiety disorders and depression were particularly elevated. The prevalence of having one or more comorbid physical illnesses was also significantly elevated in the FS cohort, albeit to a lesser degree than was the case with comorbid mental illnesses. A history of self-harm was also more prevalent among the FS cohort. Risk of all-cause mortality was not elevated in the FS cohort, and this was also true for natural and for unnatural deaths grouped broadly. There was also no statistically significant evidence of elevated suicide risk, although power was limited by low event counts. However, we did find a significant increase in incident self-harm risk among FS cohort members. The findings in the CFS sub-cohort were very similar to those found for the whole FS cohort, with one exception: lower all-cause mortality risk.

### Comparison with published findings

Consistent with previously published studies, the FS cohort examined in our study contained three times as many women as men (Jason *et al*., 2006). There were no significant elevations in the prevalence of smoking or alcohol misuse among affected persons (Woolley *et al*., 2004). However, on entry into the study FS cohort members had a raised prevalence of comorbid mental and physical illnesses. While the existing literature on comorbid physical illness is sparse, earlier studies have reported raised prevalence of psychiatric disorders as precursors or susceptibility factors,(Ranjith, 2005) as comorbid conditions (Afari and Buchwald, 2003; Cella *et al*., 2013; Larkin and Martin, 2017; Williams *et al*., 2017) or as possible consequences of the FS itself (Daniels *et al*., 2017). Roughly half of the FS cohort investigated in our study had a prior psychiatric diagnosis and similar proportions have been reported previously (Mariman *et al*., 2013).

Several studies have investigated the longer-term prognosis for patients with CFS and have noted that full recovery is uncommon, with findings from one suggesting it may be as low as 5–7% (Cairns and Hotopf, 2005). Few have an estimated risk of premature death or cause-specific mortality,(Jason *et al*., 2006) and findings are inconsistent with each study having key limitations (Smith *et al*., 2006; Jason *et al*., 2011; McManimen *et al*., 2016; Roberts *et al*., 2016). One previous study found that lifetime comorbid depression was linked with increased suicide risk among fatigued patients, but the risk elevation was not maintained when the analysis was restricted to those who met the full criteria for a CFS diagnosis (Smith *et al*., 2006). Others have reported elevated suicide risk, but conclusions were hampered in those studies by small event counts (McManimen *et al*., 2016; Roberts *et al*., 2016) or results based on crude ‘age at death’ comparisons with the general population (Jason *et al*., 2006; McManimen *et al*., 2016). Findings on all-cause mortality have been mixed; some investigators have reported elevated risks (Jason *et al*., 2011; McManimen *et al*., 2016), but another study actually reported a reduced risk among those diagnosed with CFS, although, due to the small sample size, the result was not statistically significant (Smith *et al*., 2006). Although we did not find any significant elevation or reduction in risk of either natural or unnatural death (including suicide) linked with broadly classified FSs, we did find significantly lower all-cause mortality risk in the CFS sub-cohort *v.* its respective comparison sub-cohort. We also found an increase in the proportion of patients with a history of self-harm and a significant increase in subsequent self-harm risk, but we were unable to find any published studies to corroborate or contradict this finding. We found no evidence of higher or lower suicide risk among people diagnosed with CFS, but the study wasn't adequately powered to do so.

### Strengths and limitations

This large primary care cohort provides strong evidence on mortality risk among FS patients. In previous studies, the analytical approaches were less sophisticated and robust than our matched cohort design, and the numbers of patients and events were too small to draw definitive conclusions (Jason *et al*., 2006; McManimen *et al*., 2016; Roberts *et al*., 2016). Our investigation included a large number of individuals and the study design eliminated or reduced some important sources of bias by matching patients on potential confounders such as age, gender and registered general practice. We elected not to adjust for comorbid mental illness to preclude unduly attenuating the observed association by adjusting for factors that could lie on the causal path between FS onset and subsequent adverse outcome. A key advantage of using the CPRD was its capacity for augmenting primary care records with linkage to external data sources on secondary care events and specific causes of death (McDonald *et al*., 2018).

Our investigation had a number of potential misclassification issues, most notably when attempting to delineate a definitive cohort of patients diagnosed with CFS or ME. To mitigate issues pertaining to terminology, diagnostic uncertainty and probable variation in GP coding practice, we opted to combine all FSs together for our primary analyses, and then conduct a sub-cohort analysis on patients that received a definitive diagnosis of CFS or ME. We also acknowledge the potential for surveillance bias in the detection and diagnosis of comorbidities as GPs attempt to locate the source of fatigue, resulting in greater levels of general practice attendance, probing and testing.

### Clinical implications

Despite the raised prevalence values observed for both mental and physical illnesses among persons diagnosed with FSs, our large cohort study did not discern any influence on the risk of all-cause mortality or cause-specific mortality, including suicide. This is reassuring. The raised prevalence of both physical and mental health comorbidities suggests that clinicians should take care to ensure a primary diagnosis is not missed. Assuming true comorbidity, the complex interrelationship with psychiatric conditions in particular requires further investigation using developing resources such as biobanks, which, in due course, should facilitate examination of gene-environment interactions and pathophysiology to provide answers to some of the questions raised here and in previous investigations. Although this study did not find an elevated risk of premature death from all causes or from suicide among persons with FSs, the raised prevalence of mental illness and of non-fatal self-harm risk observed indicates a need for enhanced surveillance, assessment and management of these conditions. Clinicians whose patients with FSs have self-harmed should also be aware of the following clinical guidelines: NICE, 2011; Hawton *et al*., 2016.

## Supplementary material

For supplementary material accompanying this paper visit https://doi.org/10.1017/S0033291719001065. click here to view supplementary material

## Approvals

This study is based on data from the CRPD obtained under licence from the UK MHRA. The data are provided by patients and collected by the NHS as part of their care and support. The study was approved by the independent scientific advisory committee (ISAC) for CRPD research (protocol number: 16_050R). Mortality data from the ONS^©^ (2018) and inpatient secondary-care HES^©^ (2018) were re-used with the permission of The Health & Social Care Information Centre. All rights reserved. The interpretation and conclusions contained in this study are those of the authors alone.

## Data sharing

Read and ICD-10 codes used in this study are published on clinicalcodes.org. Electronic health records are, by definition, considered ‘sensitive’ data in the UK by the Data Protection Act and cannot be shared via public deposition because of information governance restriction in place to protect patient confidentiality. Access to data is available only once approval has been obtained through the individual constituent entities controlling access to the data. The primary care data can be requested via application to the CRPD (https://www.cprd.com); secondary care data can be requested via application to the HES from the UK Health and Social Care Information Centre (http://www.hscic.gov.uk/hesdata); and mortality data are available by application to the UK ONS (http://www.ons.gov.uk/ons/index.html).

## Appendix

**Table A1.** Read codes used to identify patients with fatigue syndrome diagnoses

Read code	Description	CFS/ME
1684.13	C/O – postviral syndrome	0
8Q1..00	Activity management for chronic fatigue syndrome	1
8Q1..11	Activity management for myalgic encephalopathy	1
E205.00	Neurasthenia – nervous debility	0
Eu46000	[X]Neurasthenia	0
Eu46011	[X]Fatigue syndrome	0
Eu46y14	[X]Psychasthenia	0
Eu46y15	[X]Psychasthenia neurosis	0
F03y.12	Myalgic encephalomyelitis	1
F286.00	Chronic fatigue syndrome	1
F286.11	CFS – Chronic fatigue syndrome	1
F286.12	Post-viral fatigue syndrome	0
F286.13	PVFS – Postviral fatigue syn	0
F286.14	Post-viral fatigue syndrome	0
F286.15	Myalgic encephalomyelitis	1
F286.16	ME – Myalgic encephalomyelitis	1
F286000	Mild chronic fatigue syndrome	1
F286100	Moderate chronic fatigue syndrome	1
F286200	Severe chronic fatigue syndrome	1
R007400	[D]Postviral (asthenic) syndrome	0
R007411	[D]Post viral debility	0
