# Supplementary material for: Prevalence of comorbid mental and physical illnesses and risks for self-harm and premature death among primary care patients diagnosed with fatigue syndromes
Source: Psychol Med. 2019 May 27;50(7):1156–63. doi: 10.1017/S0033291719001065 (PMC7253619; doi:10.1017/S0033291719001065)
Supplement: Supplementary file 1 [file S0033291719001065sup001.docx]

| **Table S1. Comparison of baseline prevalence of risk factors and co-morbidities in the CFS sub-cohort vs. its matched CFS-free comparison sub-cohort** | | | | | | | |
| --- | --- | --- | --- | --- | --- | --- | --- |
|  | |  |  |  |  |  |  |
|  |  | **CFS sub-cohort** | | **CFS-free comparison**  **sub-cohort** | |  |  |
|  |  | (*N* = 4486) | | (*N* = 89,699) | |  |  |
|  |  | ***n*** | **%** | ***n*** | **%** | **PR** | **95% CI** |
|  | |  |  |  |  |  |  |
|  | |  |  |  |  |  |  |
| Co-morbid mental illnesses: | |  |  |  |  |  |  |
|  | Schizophrenia spectrum ƚ | 43 | 1.0 | 613 | 0.7 | 1.40 | (1.03, 1.91) |
|  | Bipolar disorder ƚ | 44 | 1.0 | 507 | 0.6 | 1.73 | (1.27, 2.36) |
|  | Depression ƚ | 1856 | 41.4 | 16,432 | 18.3 | 2.26 | (2.15, 2.37) |
|  | Anxiety disorder ƚ | 1816 | 40.5 | 16,037 | 17.9 | 2.26 | (2.16, 2.38) |
|  | Eating disorder ƚ | 120 | 2.7 | 1029 | 1.1 | 2.33 | (1.93, 2.82) |
|  | Personality disorder ƚ | 72 | 1.6 | 417 | 0.5 | 3.45 | (2.69, 4.43) |
|  | Any of the above diagnostic groups ƚ | 2678 | 59.7 | 25,911 | 28.9 | 2.07 | (1.99, 2.15) |
|  | |  |  |  |  |  |  |
| Psychotropic medication prescriptions: | |  |  |  |  |  |  |
|  | Anti-depressant drugs ƚ | 2998 | 66.8 | 24,286 | 27.1 | 2.47 | (2.38, 2.56) |
|  | Anti-psychotic drugs ƚ | 1163 | 25.9 | 10,452 | 11.7 | 2.22 | (2.09, 2.36) |
|  | Anxiolitics and hypnotics ƚ | 1710 | 38.1 | 16,277 | 18.1 | 2.10 | (2.00, 2.21) |
|  | Any of the above medication types ƚ | 3347 | 74.6 | 33,755 | 37.6 | 1.98 | (1.91, 2.05) |
|  | |  |  |  |  |  |  |
| History of self-harm ƚ | | 317 | 7.1 | 3514 | 3.9 | 1.80 | (1.61, 2.02) |
|  | |  |  |  |  |  |  |
| History of alcohol misuse/dependence | | 60 | 1.3 | 1129 | 1.3 | 1.06 | (0.82, 1.38) |
|  | |  |  |  |  |  |  |
| Smoking history: * | |  |  |  |  |  |  |
|  | Current/ever | 1780 | 39.7 | 36,862 | 41.1 | 0.94 | (0.90, 0.99) |
|  | Never | 2662 | 59.3 | 49,520 | 55.2 |  |  |
|  | Unknown | 44 | 1.0 | 3317 | 3.7 |  |  |
|  | |  |  |  |  |  |  |
| Co-morbid physical illness ƚ | | 1575 | 35.1 | 22,308 | 24.9 | 1.41 | (1.34, 1.49) |
|  | |  |  |  |  |  |  |
| - PR: Prevalence ratio; CI: Confidence interval - Physical comorbidity determined via the Charlson index - ƚ denotes a PR that is significantly greater than 1 - * denotes a PR that is significantly less than 1 | | | | | | | |

| **Table S2. Comparison of baseline prevalence of physical comorbidities in the CFS sub-cohort vs. its matched CFS-free comparison sub-cohort** | | | | | | |
| --- | --- | --- | --- | --- | --- | --- |
|  |  | |  | |  |  |
|  | **CFS**  **sub-cohort** | | **CFS-free**  **comparison**  **sub-cohort** | |  |  |
|  | (*N* = 4486) | | (*N* = 89,699) | |  |  |
| **Charlson condition** | ***n*** | **%** | ***n*** | **%** | **PR** | **95% CI** |
|  |  |  |  |  |  |  |
|  |  |  |  |  |  |  |
| AIDS | <5 | - | 15 | 0.0 | - | - |
| Cancer | 113 | 2.5 | 2333 | 2.6 | 0.97 | (0.80, 1.17) |
| Cerebrovascular disease ƚ | 59 | 1.3 | 796 | 0.9 | 1.48 | (1.14, 1.93) |
| Chronic pulmonary disease ƚ | 1153 | 25.7 | 15,442 | 17.2 | 1.49 | (1.41, 1.59) |
| Congestive heart disease | 19 | 0.4 | 326 | 0.4 | 1.16 | (0.73, 1.85) |
| Dementia | <5 | - | 97 | 0.1 | - | - |
| Diabetes | 131 | 2.9 | 2389 | 2.7 | 1.10 | (0.92, 1.31) |
| Diabetes with complications ƚ | 36 | 0.8 | 485 | 0.5 | 1.48 | (1.06, 2.08) |
| Hemiplegia ƚ | 20 | 0.4 | 217 | 0.2 | 1.84 | (1.17, 2.91) |
| Metastatic tumour | 11 | 0.2 | 283 | 0.3 | 0.78 | (0.43, 1.42) |
| Liver disease ƚ | 27 | 0.6 | 318 | 0.4 | 1.70 | (1.15, 2.51) |
| Myocardial infarction | 27 | 0.6 | 601 | 0.7 | 0.90 | (0.61, 1.32) |
| Peptic ulcer disease ƚ | 100 | 2.2 | 1156 | 1.3 | 1.73 | (1.41, 2.12) |
| Peripheral vascular disease | 31 | 0.7 | 483 | 0.5 | 1.28 | (0.89, 1.84) |
| Renal disease ƚ | 86 | 1.9 | 1172 | 1.3 | 1.47 | (1.18, 1.82) |
| Rheumatological disease ƚ | 110 | 2.5 | 1134 | 1.3 | 1.94 | (1.59, 2.36) |
|  |  |  |  |  |  |  |
| - PR: Prevalence ratio; CI: Confidence interval - ƚ denotes a PR that is significantly greater than 1 - * denotes a PR that is significantly less than 1 - Values omitted for AIDS and dementia due to small numbers. - Mild and moderate liver disease combined in a single category due to small numbers. | | | | | | |

| **Table S3. Hazard ratios comparing risks of premature mortality and non-fatal self-harm in the**  **CFS sub-cohort vs. its matched CFS-free comparison sub-cohort** | | | | | |
| --- | --- | --- | --- | --- | --- |
|  |  | |  | |  |
|  | **CFS**  **sub-cohort** | | **CFS-free**  **comparison**  **sub-cohort** | |  |
|  | (*N* = 4486) | | (*N* = 89,699) | |  |
|  |  | |  | |  |
|  | ***n*** | **Rate / 1000 PY** | ***n*** | **Rate / 1000 PY** | **HR (95% CI)** |
|  |  |  |  |  |  |
|  |  |  |  |  |  |
| All deaths * | 67 | 2.81 | 1789 | 3.74 | 0.76 (0.60, 0.97) |
| Natural deaths | 65 | 2.73 | 1702 | 3.56 | 0.78 (0.61, 1.00) |
|  |  |  |  |  |  |
| Self-harm ƚ | 79 | 3.35 | 762 | 1.60 | 2.11 (1.67, 2.65) |
|  |  |  |  |  |  |
| - HR = Hazard ratio - HRs adjusted for alcohol misuse and smoking status. - ƚ denotes a HR that is significantly greater than 1 - * denotes a HR that is significantly less than 1 - Results for unnatural deaths and suicide omitted due to restrictions on reporting small numbers. | | | | | |
